# Paclitaxel induces acute pain via directly activating toll like receptor 4

**DOI:** 10.1186/s12990-015-0005-6

**Published:** 2015-03-11

**Authors:** Xisheng Yan, Dylan W Maixner, Ruchi Yadav, Mei Gao, Pei Li, Michael G Bartlett, Han-Rong Weng

**Affiliations:** Department of Pharmaceutical and Biomedical Sciences, The University of Georgia College of Pharmacy, 250 West Green Street, Athens, 30602 GA USA; Department of Cardiovascular Medicine, The Third Hospital of Wuhan, Wuhan, 430060 Hubei Province China

**Keywords:** Taxol, Nociception, DRG, Neuroinflammation, EPSC, Burrowing behavior

## Abstract

Paclitaxel, a powerful anti-neoplastic drug, often causes pathological pain, which significantly reduces the quality of life in patients. Paclitaxel-induced pain includes pain that occurs immediately after paclitaxel treatment (paclitaxel-associated acute pain syndrome, P-APS) and pain that persists for weeks to years after cessation of paclitaxel treatment (paclitaxel induced chronic neuropathic pain). Mechanisms underlying P-APS remain unknown. In this study, we found that paclitaxel causes acute pain in rodents in a dose-dependent manner. The paclitaxel-induced acute pain occurs within 2 hrs after a single intravenous injection of paclitaxel. This is accompanied by low levels of paclitaxel penetrating into the cerebral spinal fluid and spinal dorsal horn. We demonstrated that an intrathecal injection of paclitaxel induces mechanical allodynia in a dose-dependent manner. Paclitaxel causes activation of toll like receptor 4 (TLR4) in the spinal dorsal horn and dorsal root ganglions. Through activating TLR4, paclitaxel increases glutamatergic synaptic activities and reduces glial glutamate transporter activities in the dorsal horn. Activations of TLR4 are necessary in the genesis of paclitaxel-induced acute pain. The cellular and molecular signaling pathways revealed in this study could provide rationales for the development of analgesics and management strategies for P-APS in patients.

## Introduction

Paclitaxel (taxol) is a first-line chemotherapeutic-agent used for the treatment of many types of cancers. Patients receiving taxol treatment often develop pathological pain, which significantly reduces their quality of life and hampers the use of this otherwise life-saving chemotherapy in the clinic. Pathological pain induced by taxol in patients includes pain that occurs immediately after taxol treatment (known as paclitaxel-associated acute pain syndrome, P-APS) [1,2], and pain that persists for weeks to years after cessation of paclitaxel treatment (known as paclitaxel induced chronic neuropathic pain) [3,4]. P-APS is a significant morbidity in patients [1,2]. Currently, there is no proven standard of care for the prevention or treatment of P-APS and mechanisms by which paclitaxel induces P-APS are not known. The current understanding of mechanisms underlying taxol-induced pathological pain in animal models is mainly based on studies that examine pathological changes days after cessation of repeated taxol treatments. Little is known about the direct and immediate action induced by taxol on the pain signaling system in animal models.

It has been known since 1993 that treatment of paclitaxel can immediately induce pain symptom in patients [5,6]. More recently such pain was defined as P-APS [1,2]. P-APS is characterized by its early onset (occurring within 1–3 days after drug administration) and short lasting period (usually resolving within 7 days) [1,2]. To date, no studies have been conducted to examine mechanisms related to the taxol induced acute pain in animal models. Understanding such mechanisms could provide rationales for the development of analgesics and treatments for P-APS.

Paclitaxel has lipopolysaccharide (LPS)-mimetic activity causing the activation of toll like receptor 4 (TLR4) and inducing the synthesis and release of several proinflammatory cytokines including tumor necrosis factor α (TNFα) and interleukin-1β (IL-1β) in human monocytes, T-lymphocytes, ovarian cancer cell lines [7], murine peritoneal macrophages [8] and breast cancer cell lines [9]. The increased synthesis and release of TNFα and IL-1β is independent of the effects of taxol on microtubules [10]. TLR4 is an innate immune pattern recognition receptor, expressed predominantly on microglia in the central nervous system (CNS) [11,12]. Activation of TLR4 is critically implicated in the development and maintenance of pathologic pain [13-15]. Paclitaxel is known for its poor penetration into the CNS even though low concentrations of paclitaxel in human cerebral spinal fluid (CSF) [16,17] and rodent spinal cord [18] and brain [19,20] have been repeatedly demonstrated. It remains unexplored whether traces of paclitaxel penetrating into the spinal cord produce any functional impacts on the spinal pain signaling system.

Excessive activation of glutamate receptors in spinal dorsal horn neurons is a hallmark mechanism of pathological pain [21,22]. Activation of glutamate receptors is governed by three key factors: the amount of glutamate released from presynaptic terminals, the function and number of postsynaptic glutamate receptors, and the function of glutamate transporters. Since glutamate is not metabolized extracellularly, the clearance of glutamate released from presynaptic terminals and maintenance of glutamate homeostasis depend on glutamate transporters [21,23,24]. Glutamate transporters up-take glutamate into the cell. Glial glutamate transporters account for more than 90% of all CNS synaptic glutamate uptake [25] and are a key part of the machinery for regulating synaptic signal transmission [23]. Two types of glial glutamate transporters [glial glutamate transporter 1 (GLT-1) and glutamate aspartate transporter (GLAST)] are present in the spinal dorsal horn [26,27]. We and others have demonstrated that downregulation of astrocytic glutamate transporter protein expression in the spinal dorsal horn is associated with neuropathic pain (chronic allodynia) induced by repeated treatments of taxol [26,28] or nerve injury [29-31]. It is unknown whether changes in glutamate released from presynaptic terminals, postsynaptic glutamate receptors, and glial glutamate transporters contribute to the development of paclitaxel-induced acute pain.

In this study, we found that intravenous or intrathecal administration of paclitaxel causes activation of TLR4 in the spinal dorsal horn and dorsal root ganglions, and TLR4 is required for paclitaxel to induce acute pain in rodents. The direct impacts induced by paclitaxel on the spinal glutamatergic synapses and glial glutamate transporters were uncovered.

## Results

### Intravenous injection of paclitaxel induces acute pain in rodents

**Figure 1:**
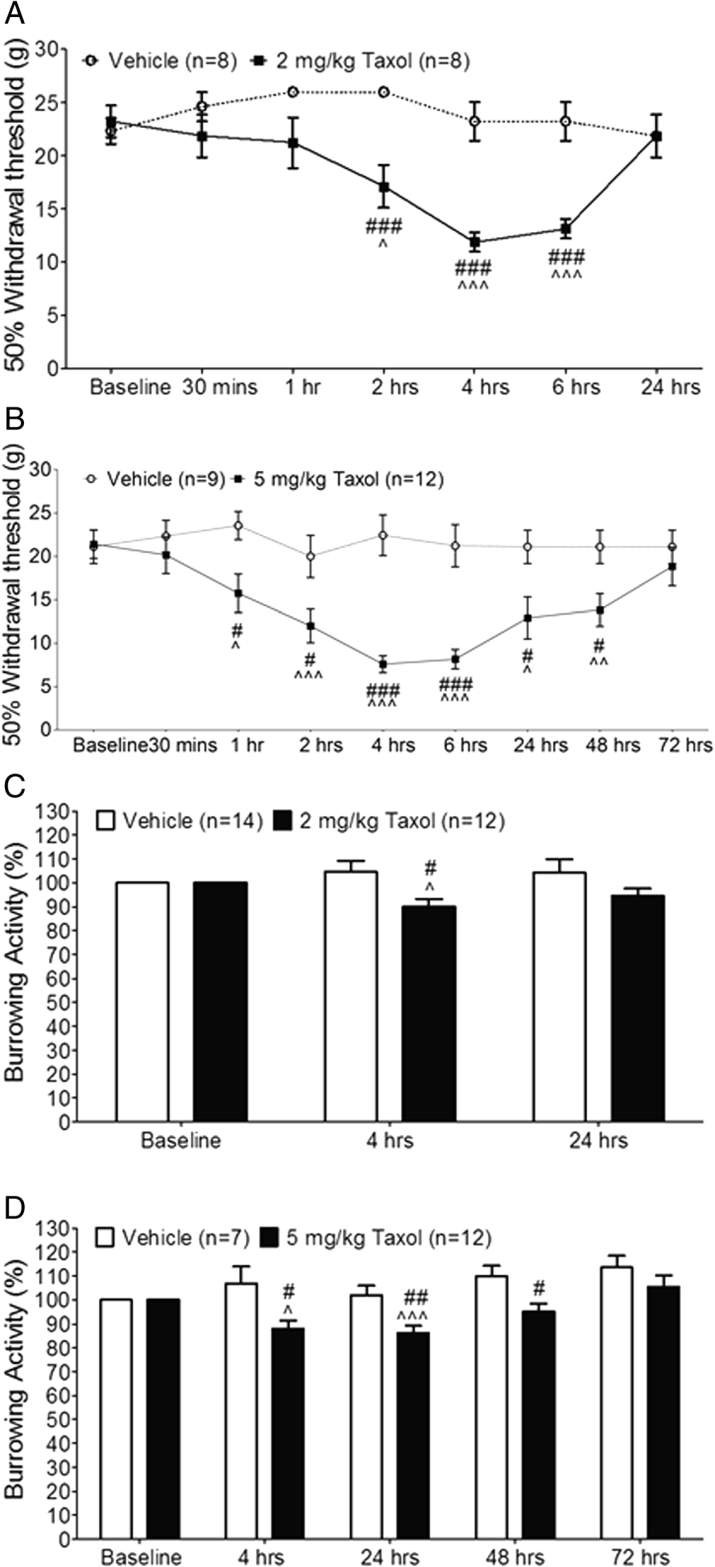
**Paclitaxel induces acute mechanical allodynia and reduces burrowing activities. (A)** and **(B)**: Mechanical thresholds of withdrawal responses in rats before (baseline) and at different time points after i.v. injection of paclitaxel at 2 mg/kg **(A)**, 5 mg/kg **(B)**, or vehicle are plotted. **(C)** and **(D)**: Burrowing activities in rats at different time points after i.v. injection of paclitaxel at 2 mg/kg **(C)**, 5 mg/kg **(D)**, or vehicle are normalized to burrowing activities collected before the i.v. injection (baseline). Comparisons between baseline and at each time point are indicated with ^ for the paclitaxel group. Comparisons between the vehicle group and the paclitaxel group at each time point are labeled with #. One symbol: *P* < 0.05; Two symbols: *P* < 0.01; Three symbols: *P* < 0.001.

To determine whether paclitaxel induces acute pain in animals, nociceptive behaviors in rats were examined after the rats were treated with paclitaxel or saline. Paclitaxel (dose: 2 mg/kg; volume: 1 ml; duration of the injection: 1 min) was given to rats via the tail vein to simulate intravenous (i.v.) administration of taxol used in the clinic. Vehicle control (1 ml) was injected into the rats in the same fashion in the control group. A single i.v. injection of taxol reduced thresholds of hind paw withdrawal responses to mechanical stimuli, which occurred within 2 hrs and peaked at 4 hrs but resolved within 24 hrs after taxol injection (Figure 1A). Meanwhile, thresholds of hind paw withdrawal responses to mechanical stimuli in rats receiving vehicle (control group) remained unchanged. This is in agreement with a previous report [32] showing that a single intraperitoneal injection (i.p.) of paclitaxel (1 mg/kg) in rats induces acute mechanical allodynia between 1 and 6 hrs after paclitaxel treatment. Furthermore, we also determined the changes of mechanical thresholds following i.v. paclitaxel injection at a higher dose (5 mg/kg). Mechanical allodynia was observed within 1 hr, peaked between 4 to 6 hrs, lasted more than 48 hrs, and ended before 72 hrs after paclitaxel injection (Figure 1B), which lasts significantly longer than that induced by paclitaxel at 2 mg/kg (Figure 1A). At the same time period, mechanical thresholds in rats receiving vehicle were not significantly altered. These data indicate that paclitaxel induces acute mechanical allodynia in a dose-dependent manner.

Recent studies have demonstrated that innate burrowing behavior can be used to measure the wellbeing of rodents. When animals are in a status of pain, burrowing behavior is reduced [33,34]. We then measured burrowing behaviors in rats treated with paclitaxel. After quantifying the burrowing behavior at baseline, rats were divided into two groups, one group receiving i.v. paclitaxel, the other receiving vehicle. We first determined burrowing behaviors in rats receiving i.v. paclitaxel (2 mg/kg) or vehicle. As mechanical allodynia induced by paclitaxel at this dose peaked at 4 hrs and disappeared at 24 hrs after paclitaxel injection, burrowing behaviors were measured at 4 and 24 hrs after the i.v. injection. In comparison with their own baseline and their counterpart in the vehicle group, the burrowing behaviors in the paclitaxel group were significantly decreased at 4 hrs, but returned to baseline by 24 hrs (Figure 1C). When we measured burrowing behaviors in rats receiving paclitaxel at 5 mg/kg (i.v.), we found that burrowing behaviors were significantly reduced at 4, 24, and 48 hrs, but recovered by 72 hrs after paclitaxel injection (Figure 1D). Taking all data in Figure 1 together, we conclude that paclitaxel induces acute pain in rats in a dose-dependent manner.

### Low levels of paclitaxel are found in the CSF and spinal dorsal horn after paclitaxel injection and intrathecal injection of paclitaxel induces acute pain in rats

**Figure 2:**
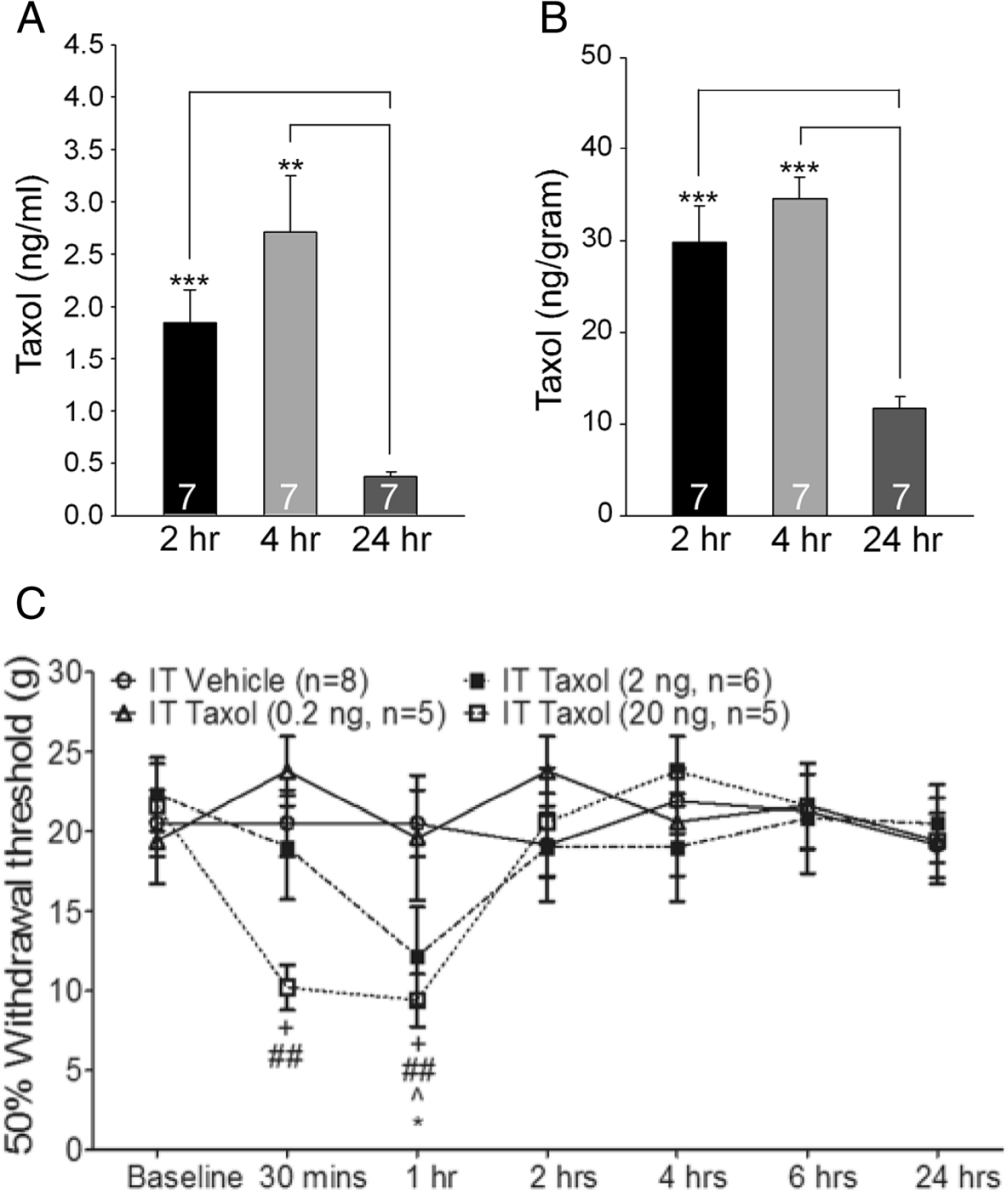
**Low levels of paclitaxel are found in the CSF and spinal dorsal horn after paclitaxel injection and intrathecal injection of paclitaxel induces acute pain in rats. (A)** and **(B)**: Concentrations of paclitaxel in the CSF **(A)** and spinal dorsal horn **(B)** in rats at 2, 4, and 24 h after i.v. injection of paclitaxel (2 mg/kg) are shown. Number of animals included in each group for the analysis is shown in each bar. **P < 0.01; ***P < 0.001. **(C)**: Mechanical thresholds of withdrawal responses in rats before (baseline) and different time points after intrathecal injection of paclitaxel at 0.2 ng, 2 ng, and 20 ng, or vehicle in different groups are plotted. Comparisons between baseline and at each time point are indicated with ^ for rats receiving 2 ng paclitaxel, and with + for rats receiving 20 ng paclitaxel. Comparisons at each time point between rats receiving vehicle, and rats receiving 2 ng paclitaxel are labeled with *, or rats receiving 20 ng paclitaxel are indicated with #. One symbol: *P* < 0.05; Two symbols: *P* < 0.01.

To investigate whether paclitaxel penetration into the spinal dorsal horn correlates to paclitaxel-induced acute pain, paclitaxel concentrations in the CSF and spinal dorsal horn of rats at three time points (2 hrs, 4 hrs and 24 hrs after i.v. injection of paclitaxel at 2 mg/kg) were analyzed using liquid chromatography tandem mass spectrometry. The 2 and 4 hrs time points corresponded to the time when allodynia occurred and the 24 hrs time point corresponded to the time when allodynia disappeared (Figure 1A). Paclitaxel concentrations reached mean levels of 1.85 to 2.70 ng/ml in the CSF (n = 7) (Figure 2A) and 29.85 to 34.55 ng/g in the spinal dorsal horn (n = 7) (Figure 2B) between 2 to 4 hrs after paclitaxel injection. These paclitaxel concentrations went down significantly to mean levels of 0.37 ng/ml in the CSF (n = 7) and 11.67 ng/ml in the spinal dorsal horn (n = 7) 24 hrs after the injection. These concentrations are consistent with data collected from human CSF [16,17], rodent spinal cord [18] and mouse brains [19,20] sampled within a similar time frame. Because the occurrence and disappearance of acute pain behaviors coincided with changes of paclitaxel concentrations in the CSF and spinal dorsal horn tissue, we proposed that paclitaxel in the CSF and spinal dorsal horn may contribute to the development of the paclitaxel induced acute pain. This was supported by the following set of experiments. We injected paclitaxel or vehicle (in a volume of 20 μl) directly into the intrathecal space through lumbar puncture in rats and examined the animal nociceptive behaviors. Three doses of paclitaxel (0.2 ng, 2 ng, and 20 ng) were used. As shown in Figure 2C, in comparison with rats injected with vehicle, rats receiving paclitaxel developed mechanical allodynia in a dose-dependent manner, which occurred within 30 min and disappeared at 2 hrs after the injection.

### Activation of TLR4 in the spinal dorsal horn and dorsal root ganglions is critically implicated in paclitaxel-induced acute pain

**Figure 3:**
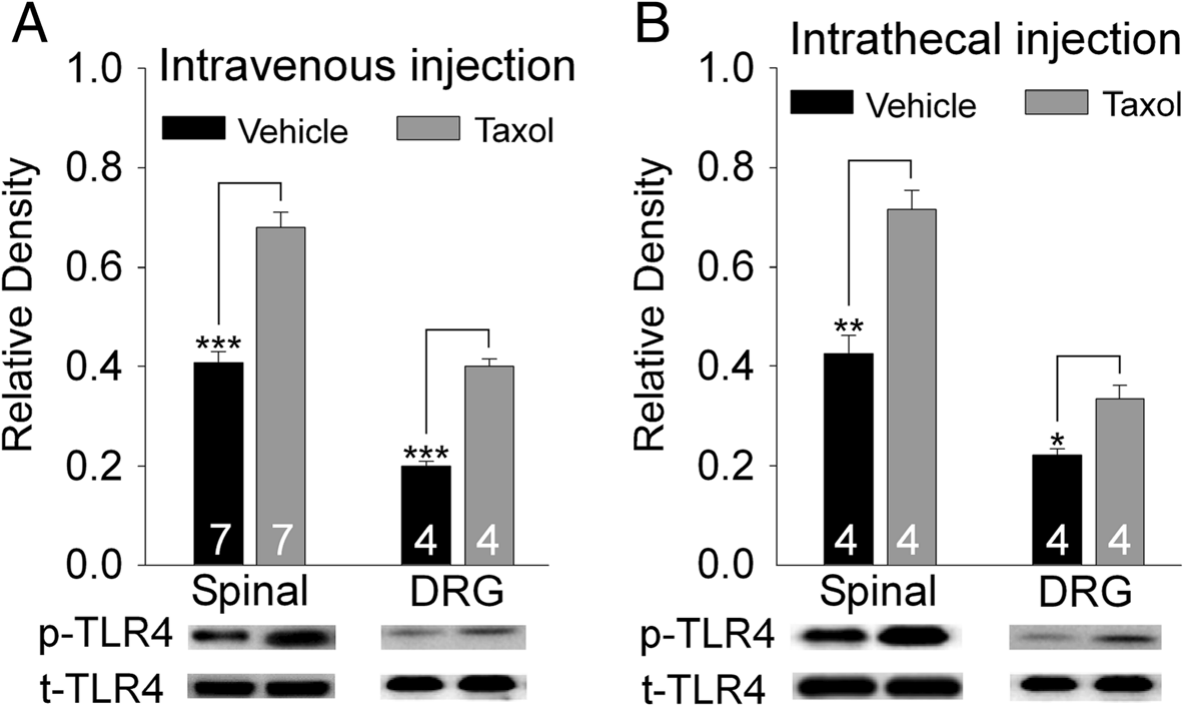
**TLR4 in the spinal dorsal horn and dorsal root ganglions are activated following intravenous or intrathecal injection of paclitaxel.** Expression of phosphorylated TLR4 (p-TLR4) and total TLR4 (t-TLR4) in the spinal dorsal horn and dorsal root ganglions of L4-L5 spinal segments 4 hrs after i.v. injection of taxol (2 mg/kg) or vehicle are shown in **(A)**. Expression of phosphorylated TLR4 (p-TLR4) and total TLR4 (t-TLR4) in the spinal dorsal horn and dorsal root ganglions of L4-L5 spinal segments 1 hr after intrathecal injection of taxol (20 ng) or vehicle are shown in **(B)**. Number of animals included in each group for the analysis is indicated in each bar. *P < 0.05; **P < 0.01; ***P < 0.001.

It is known that paclitaxel has LPS-mimetic activity causing activation of TLR4 [7,35,36], and activation of TLR4 causes TLR4 tyrosine phosphorylation (p-TLR4) [37,38]. Using Western blot, we determined if TLR4 receptors are activated following paclitaxel i.v. injection by examining p-TLR4 levels in the spinal dorsal horn and dorsal root ganglions at the L4-L5 segments. We found that in comparison with rats treated with i.v. vehicle injection, expressions of p-TLR4 in the spinal dorsal horn and dorsal root ganglions were increased, whereas total TLR4 (t-TLR4) remained unchanged 4 hrs after i.v. injection of taxol (2 mg/kg) (Figure 3). We then examined levels of p-TLR4 in the spinal dorsal horn and dorsal root ganglions at the L4-L5 spinal segments in rats 1 hr after paclitaxel (20 ng) or vehicle (in a volume of 20 μl) was injected into the intrathecal space through lumbar puncture. We found that in comparison with rats receiving vehicle treatment, levels of p-TLR4 in the spinal dorsal horn and dorsal root ganglions were increased in rats treated with intrathecal (i.t.) injection of paclitaxel. At the same time, no difference of t-TLR4 levels was found between paclitaxel treated and vehicle-treated groups. These data indicate that TLR4 at the spinal dorsal horn and dorsal root ganglions is activated following i.v. or i.t. injection of paclitaxel.

**Figure 4:**
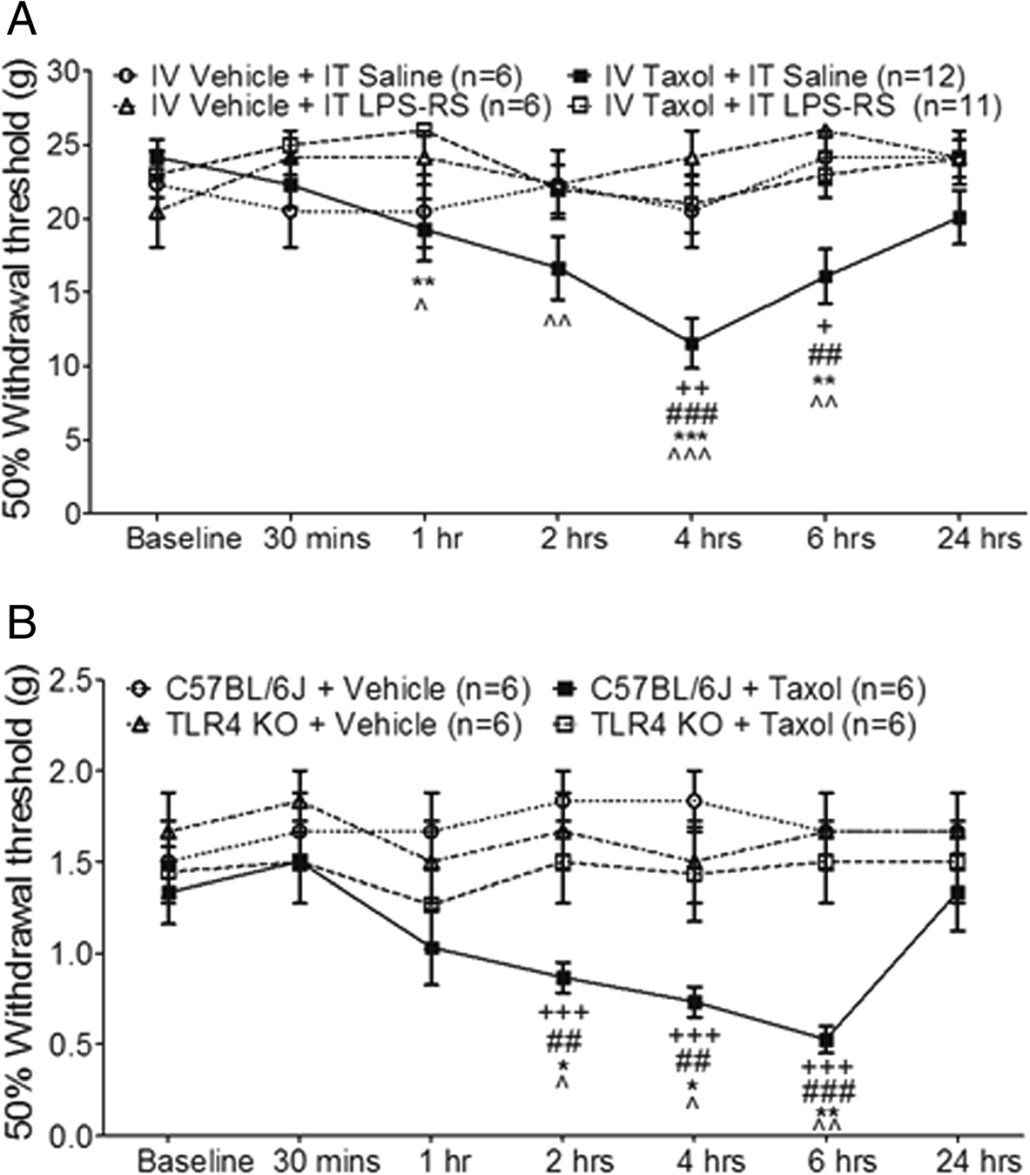
**Activation of TLR4 in the spinal dorsal horn is critically implicated in the paclitaxel-induced acute allodynia. (A)**: Line plots show summaries of the mechanical thresholds of hind paw withdrawal responses (mean ± S.E.) in 4 groups of rats. Baseline indicates the baseline measurement before animals received any treatments. Taxol (2 mg/kg) or vehicle was given to rats through the tail vein. LPS-RS or saline was applied into the lumbar enlargement through an intrathecal catheter immediately before and at 3 h after i.v. injection of taxol or vehicle. Comparisons between baseline and at each time point are indicated with v for the IV Taxol + IT saline group. Comparisons between the IV Taxol + IT saline group and the IV Taxol + IT LPS-RS group are labeled with *. Comparisons between the IV Taxol + IT saline group and the IV vehicle + IT LPS-RS group are labeled with #. Comparisons between the IV taxol + IT saline group and the IV vehicle + IT saline group are labeled with +. **(B)**: Line plots show summaries of the mechanical thresholds of hind paw withdrawal responses (mean ± S.E.) in 4 groups of mice. Taxol (2 mg/kg) or vehicle was injected to mice intraperitoneally. Comparisons between baseline and at each time point are indicated with ^ for the C57BL/6J (wild-type) + Taxol group. Comparisons between the C57BL/6J + Taxol group and the C57BL/6J + vehicle group are labeled with +. Comparisons between the C57BL/6J + Taxol group and the TLR4 Knockout (KO) + taxol group are labeled with *. Comparisons between the TLR4 Knockout (KO) + vehicle group and the C57BL/6J+ taxol group are labeled with #. One symbol: *P* < 0.05; Two symbols: *P* < 0.01; Three symbols: *P* < 0.001.

We next determined whether activation of TLR4 contributes to the development of acute pain induced by paclitaxel. Rats with pre-implanted intrathecal catheters were randomly assigned into 4 groups: paclitaxel + i.t. TLR4 antagonist group, paclitaxel + i.t. saline group, vehicle + i.t. TLR4 antagonist group, and vehicle + i.t. saline group. The TLR4 antagonist lipopolysaccharide-RS (LPS-RS, 40 μg in a volume of 10 μl) was injected into the rats through the implanted catheter immediately before and 3 hrs after the i.v. injection of taxol (2 mg/kg, in a volume of 1 ml) in the paclitaxel + i.t. TLR4 antagonist group. The same type of LPS-RS treatment was applied to rats receiving the i.v. injection of vehicle (1 ml) in the vehicle + i.t. TLR4 antagonist group. Intrathecal injection of saline was applied to the rats in the paclitaxel + i.t. saline group and the vehicle + i.t. saline group in the same fashion. The dose of LPS-RS was based on previous studies [39-41]. As shown in Figure 4A, mechanical allodynia was basically abolished in the paclitaxel + i.t. TLR4 antagonist group in comparison with the paclitaxel + i.t. saline group. Meanwhile nociceptive behaviors in the vehicle + i.t. TLR4 antagonist group, and vehicle + i.t. saline group were not significantly altered during the same observation period. These data indicate that activation of TLR4 is a critical event in the genesis of taxol induced acute pain.

To further confirm the role of TLR4 in paclitaxel-induced acute pain, the effects of paclitaxel on nociceptive behaviors in TLR4 knockout mice and wild-type (C57BL/6J) mice were examined (Figure 4B). Similar to those observed in rats (Figure 1), mechanical thresholds of hind paw withdrawal responses in wild-type mice began to drop within 2 hrs and peaked at 6 hrs and resolved by 24 hrs after receiving paclitaxel injection (2 mg/kg, in a volume of 0.25 ml). Mechanical thresholds in mice receiving vehicle treatments were not significantly altered in the same period. Prior to any treatments, TLR4 knockout mice had similar mechanical thresholds as wild-type mice (Figure 4B). However, when paclitaxel (2 mg/kg, in a volume of 0.25 ml) or vehicle (0.25 ml) was i.p. injected to TLR4 knockout mice, mechanical thresholds remained unchanged in both groups (Figure 4B). These data further confirm our conclusion that TLR4 is critically implicated in the paclitaxel-induced acute pain.

### Paclitaxel increases the release of glutamate from presynaptic terminals and the activity of glutamate receptors at postsynaptic neurons in the spinal dorsal horn

**Figure 5:**
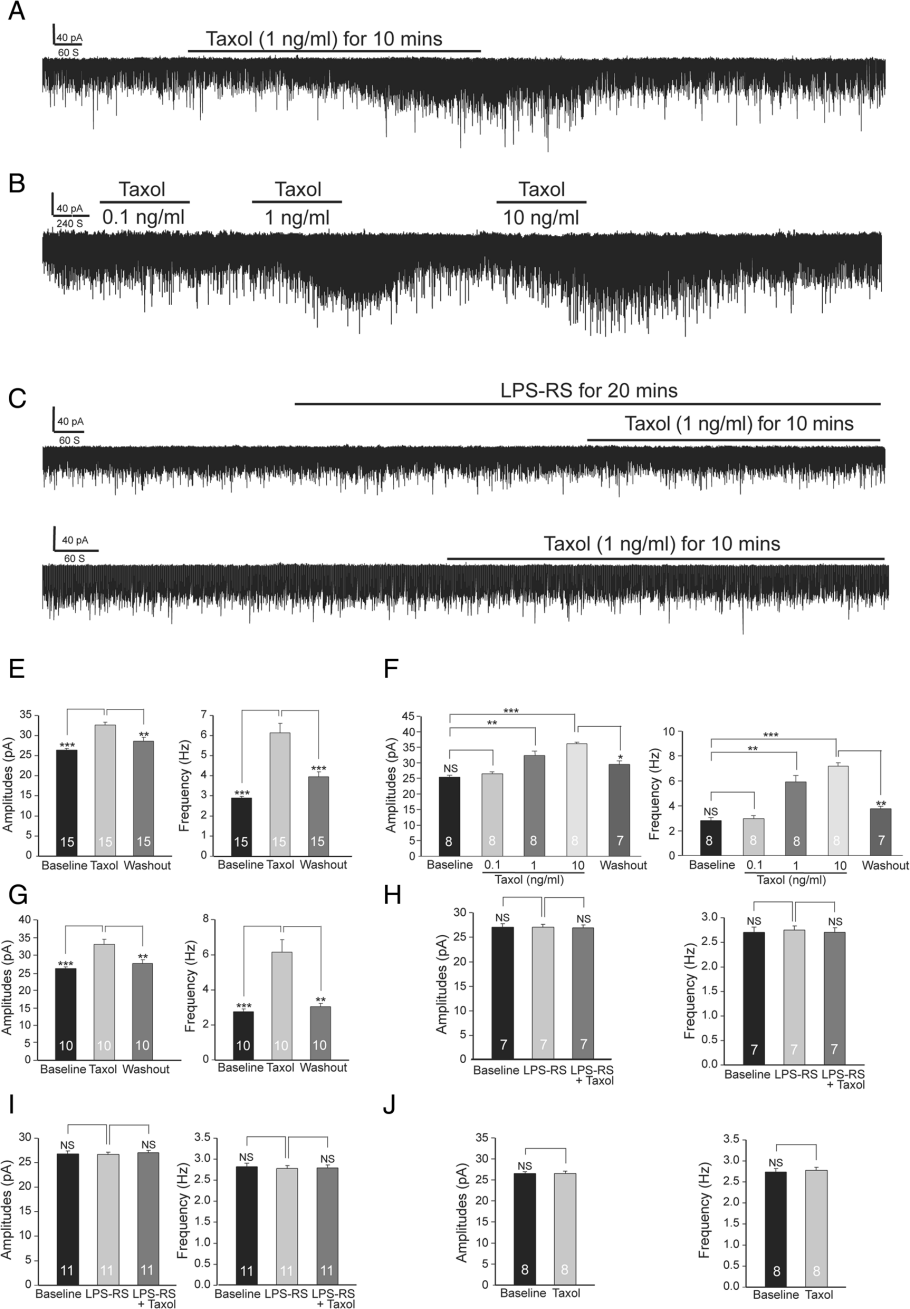
**Paclitaxel increases the release of glutamate from presynaptic terminals and activities of glutamate receptors at postsynaptic neurons in the spinal dorsal horn through activation of TLR4.** Raw data show that bath-perfusion of paclitaxel significantly increased both the frequency and amplitudes of mEPSCs in a dose-dependent manner in rats **(A)** and **(B)**, but these effects were abolished in rats in the presence of the TLR4 inhibitor (LPS-RS) **(C)**, and in TLR4 knockout mice **(D)**. Bar graphs show the mean (+ S.E.) frequency and amplitude of mEPSCs before, during, and after washout of paclitaxel (1 ng/ml) in rats **(E)** and wild-type mice **(G)**, as well as in the presence of LPS-RS in rats **(H)** and wild-type mice **(I)**. The mean (+ S.E.) frequency and amplitude of mEPSCs before, during, and after washout of paclitaxel at 0.1 ng/ml, 1 ng/ml, and 10 ng/ml in rats are presented in **(F)**. The mean (+ S.E.) frequency and amplitude of mEPSCs before and during paclitaxel (1 ng/ml) perfusion in TLR4 knockout mice are shown in **(J)**. Number of neurons included in each group for the analysis is shown in each bar. **P < 0.01; ***P < 0.001; NS, no statistical significance.

We next investigated the direct impact, induced by activation of spinal TLR4 upon paclitaxel administration, on glutamatergic synapses in the spinal dorsal horn because excessive activation of glutamatergic receptors in the spinal dorsal horn is one of the most critical mechanisms leading to abnormal neuronal activation in the pain signaling pathway in pathological pain conditions [24,42-44]. To study the direct action of paclitaxel on glutamatergic synaptic activities in the spinal dorsal horn, we determined the effects of paclitaxel on glutamate release from presynaptic terminals and postsynaptic glutamate receptor activities in the spinal dorsal horn. Analysis of miniature excitatory postsynaptic currents (mEPSCs) is a conventional approach to identify changes of glutamatergic synapses at the pre- and post- synaptic levels. An increase in mEPSC frequencies indicates an increase in the presynaptic transmitter release probability, whereas an increase in mEPSC amplitudes indicates an increase in the post-synaptic receptor activities [45,46]. Neurons receiving monosynaptic input from peripheral A and C fibers in the superficial spinal dorsal horn (lamina I and outer lamina II) [44,47] were recorded. Glutamatergic mEPSCs in dorsal horn neurons of rats and mice were recorded before and after bath perfusion of paclitaxel (1 ng/ml, the low end concentration of paclitaxel found in the CSF 4 hrs after i.v. injection of taxol). Paclitaxel perfusion significantly increased glutamatergic mEPSC frequencies from 2.88 ± 0.08 to 6.14 ± 0.47 Hz (n = 15, P < 0.001) and amplitudes from 26.32 ± 0.47 to 32.60 ± 0.71 pA (n = 15, P < 0.001) in slices of rats (Figure 5A, and E). Similarly, mEPSCs frequencies and amplitudes recorded from mice were significantly increased (n = 10, P < 0.001) after paclitaxel (1 ng/ml) was added into the bath (Figure 5G). Furthermore, in the other 8 cells recorded from rats, we examined their responses to paclitaxel perfusion at three concentrations (0.1 ng/ml, 1 ng/ml, and 10 ng/ml). As shown in Figure 5B and F, paclitaxel increased the mEPSC frequencies and amplitudes in a dose-dependent manner. These data indicate that the paclitaxel-induced acute pain results, at least in part, from increased release of glutamate from presynaptic terminals, and increased postsynaptic glutamate receptor functions in the spinal dorsal horn.

### Frequencies and amplitudes of mEPSCs are increased by paclitaxel in spinal dorsal horn neurons through activation of TLR4

To determine whether activation of TLR4 is involved in the increased glutamatergic synaptic activities induced by paclitaxel in the spinal dorsal horn, we recorded glutamatergic mEPSCs from neurons that received monosynaptic input from the primary afferents in the superficial spinal dorsal horn in rats and mice. After recording baseline mEPSCs, we bath-perfused the TLR4 antagonist (LPS-RS, 2 μg/ml). LPS-RS did not change either mEPSC frequencies or amplitudes in rats (Figure 5C and H) and mice (Figure 5I). In the presence of LPS-RS, further addition of paclitaxel (1 ng/ml) did not induce changes in mEPSC frequencies or amplitudes in rats (Figure 5C and H) and wild-type mice (Figure 5I), which is in contrast with data collected in the absence of LPS-RS. Further, when we recorded mEPSCs from spinal slices obtained from TLR4 knockout mice, mEPSC frequencies and amplitudes were not altered by bath-application of paclitaxel (1 ng/ml) (Figure 5D and J). These data indicate that glutamatergic synaptic activities in normal conditions are not under the control of TLR4 activities, and paclitaxel increases the release of glutamate from presynaptic terminals and function of glutamate receptors at the postsynaptic neurons in the spinal dorsal horn through activation of TLR4.

### Dysfunction of glial glutamate transporters (GTs) contributes to the development of the paclitaxel-induced acute pain

**Figure 6:**
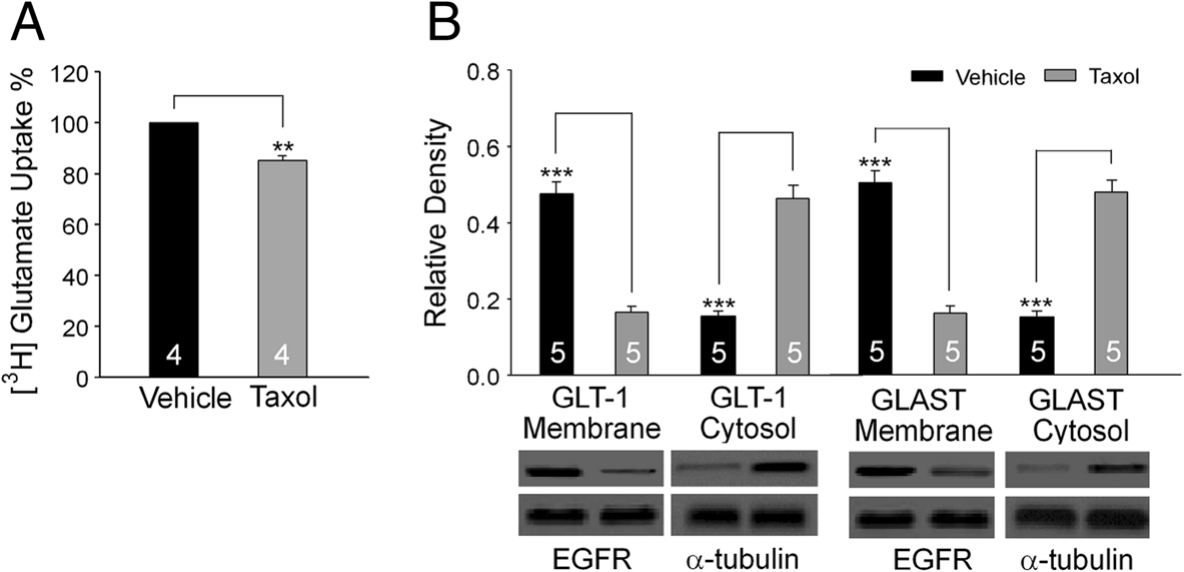
**Dysfunction of glial glutamate transporters contributes to the development of paclitaxel-induced acute allodynia. (A)**: Bar graphs show percentage changes of glutamate uptake in the spinal dorsal horn after paclitaxel (2 ng/ml) was applied onto the intact spinal cord for 30 min. **(B)**: Paclitaxel reduces GLT-1 and GLAST protein expressions in the cell surface. Samples of GLT-1 and GLAST protein expressions in the cell surface (membrane) and cytosol in spinal dorsal horn slices treated with paclitaxel and vehicle for 15 minutes are shown. Bar graphs show the mean (+S.E.) relative density of GLT-1 and GLAST in the plasma membrane to EGFR, and the mean (+S.E.) relative density of GLT-1 and GLAST in the cytosol to α-tubulin in each group. Number of animals included in each group for the analysis is shown in each bar. ***P < 0.001.

We previously demonstrated that the function of glutamate transporters is a key factor regulating activation of glutamate receptors in the spinal dorsal horn [48,49]. To determine if glutamate transporter functions are altered by paclitaxel, we simulated the topical effects induced by paclitaxel in the CSF on spinal glutamate transporter activities in the intact spinal cord. After exposing the L4-L5 spinal segments with the dura open and the pia intact in anesthetized rats, we topically placed a piece of cotton, which was soaked with taxol (concentration: 2 ng/ml) in artificial cerebrospinal fluid (aCSF) at 35°C, onto the L4-L5 region for 30 min. This taxol concentration is within the range of taxol concentrations in the CSF and spinal dorsal horn tissue in rats receiving taxol (2 mg/kg) injection (Figure 2). Synaptosome preparations were prepared from the L4-L5 spinal dorsal horn immediately after the taxol treatment and incubated with [^3^H]L-glutamate. The glutamate uptake was determined by measuring the radioactivity of [^3^H]L-glutamate in the synaptosome preparations [50,51]. In comparison with the control (n = 4) treated with vehicles, the synaptosome preparations obtained from rats receiving the topical paclitaxel treatment had a 14.95 ± 2.13% (n = 4. P < 0.01) reduction of glutamate uptake activities (Figure 6A). These results indicate that dysfunction of GTs in the spinal dorsal horn contributes to the development of paclitaxel-induced acute pain. Further, these data also provide evidence that the reduction of glutamate uptake activities in the spinal dorsal horn after paclitaxel i.v. injection is directly due to paclitaxel penetrating to the CSF and spinal cord tissue.

To determine mechanisms underlying the suppression induced by taxol on glutamate uptake activities, we analyzed trafficking of glial GTs (GLT-1 and GLAST), between the plasma membrane and cytosol in rat spinal slices of L4-L5 segments (400 μm thick) incubated for 15 min in 2 conditions: paclitaxel (1 ng/ml) in aCSF and vehicles. Compared to slices treated with vehicles, slices treated with paclitaxel had decreased protein expressions of GLT-1 and GLAST in the cell membrane but increased expressions of GLT-1 and GLAST in the cytosol (Figure 6B). The sum expression of GLT-1 in the membrane and cytosol and the sum expression of GLAST in the membrane and cytosol in the paclitaxel-treated slices were similar to their counterparts in slices treated with vehicles (n = 5; data not shown). These data indicate that internalization of GLT-1 and GLAST in the spinal dorsal horn is an important mechanism leading to a reduction of glutamate uptake induced by paclitaxel.

### Paclitaxel reduces glial glutamate transporter activities through activating TLR4

**Figure 7:**
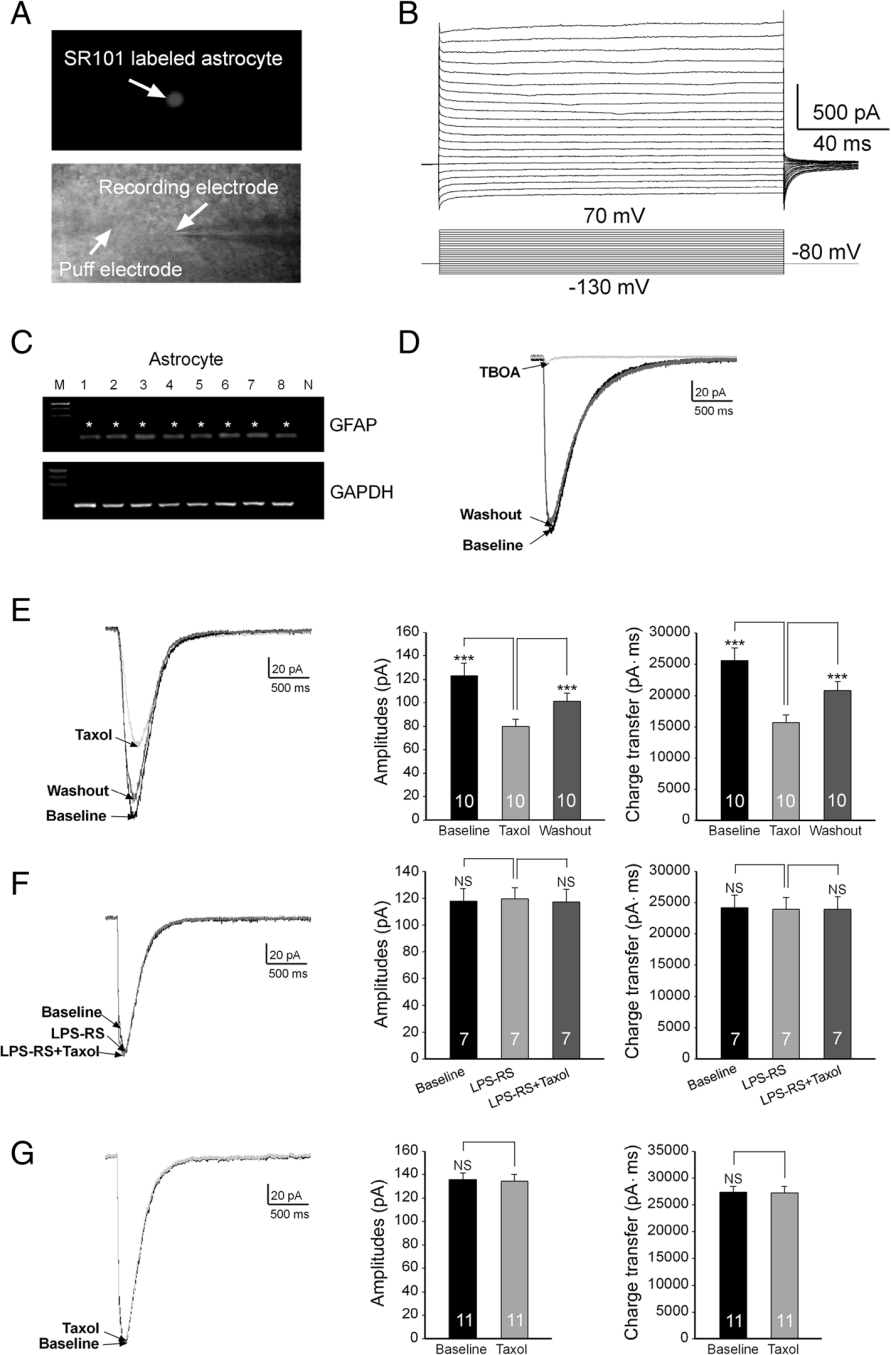
**Paclitaxel reduces glial glutamate transporter activities through activating TLR4. (A)**: An astrocyte in the mouse spinal dorsal horn was stained by the astrocyte specific dye, sulforhodomine 101 (SR101, 100 μM) (top). **(B)**: Inward and outward currents (top) in a spinal astrocyte were evoked by voltage steps (10 mV/step) from −130 mV to +70 mV (bottom), indicating a passive membrane property of astrocytes. **(C)**: Astrocytes identified by this way all (8 cells) expressed GFAP. N: Negative control. GAPDH was used as internal control. GTCs were evoked by L-glutamate (50 μM) injected onto the astrocyte through a puff-electrode (A, bottom). Such currents were almost abolished in the presence of the specific glial glutamate transporter blocker TFB-TBOA (10 μM) **(D)**. Bath-perfusion of paclitaxel (1 ng/ml) significantly reduced GTC amplitudes and charge transfers **(E)**, and these effects were abolished in the presence of the TLR4 antagonist LPS-RS (2 μg/ml) **(F)**. **(G)**: GTCs recorded from spinal slices obtained from TLR4 knockout mice were not altered by bath-perfusion of paclitaxel (1 ng/ml). Bar graphs show the mean (+S.E.) GTC amplitude and charge transfer before (baseline), during and after washout of the tested agent(s). Number of animals included in each group for the analysis is shown in each bar. ***P < 0.001; NS, no statistical significance.

To investigate how taxol suppresses glial glutamate transporter activities, we monitored glial glutamate transporter activities in real time by recording glutamate transporter currents (GTCs) from astrocytes. The uptake of glutamate by glial glutamate transporters is accompanied by the co-transport of two or three Na^+^ with one H^+^ and the countertransport of one K^+^ [52-54]. Because of the translocation of a net positive charge during each transport cycle, glutamate uptake generates a current called GTC [52-54]. The size of GTC reflects the amount of transported glutamate, which has been widely used as an effective tool to study the function of glial glutamate transporters [51,55-57]. GTCs were recorded from spinal slices of mice. Astrocytes were labeled by the astrocyte specific dye, sulforhodomine 101 (100 μM) [58] (Figure 7A), which was pressure-injected into the spinal slice through a pipette with a picospritzer [29,58,59]. The recorded cells displayed a linear IV relationship (a passive membrane property) (Figure 7B) and a low input resistance (10–20 MΩ), a membrane property characteristic of astrocytes. The accuracy of such techniques in identifying astrocytes was confirmed by single-cell RT-PCR (Figure 7C) in 8 cells. All 8 cells identified using this technique expressed the glial fibrillary acid protein (GFAP), demonstrating the reliability of this technique. GTCs were evoked by L-glutamate (50 μM) injected onto the astrocyte through a puff-electrode [51]. Such currents were almost abolished in the presence of the specific glial glutamate transporter blocker TFB-TBOA (10 μM) [60] (Figure 7D), confirming that these currents were generated from glutamate transporter activities. We found that perfusion of paclitaxel directly onto the recording bath at a concentration as low as 1 ng/ml significantly reduced GTC amplitudes by 34.11 ± 4.42% (n = 10, P < 0.001) and charge transfers by 39.10 ± 3.68% (n = 10, P < 0.001) (Figure 7E). These data confirm that glial glutamate transporter activities are reduced by paclitaxel. We next determined whether TLR4 mediates the effects induced by paclitaxel on GTCs. After recording baseline GTCs, we bath-perfused the TLR4 antagonist (LPS-RS, 2 μg/ml). Perfusion of LPS-RS did not induce changes in GTC charge transfers and amplitudes, indicating that glial glutamate transporter activities are not controlled by TLR4 activation under normal conditions. In the presence of LPS-RS, further addition of paclitaxel (1 ng/ml) into the recording bath did not alter GTC charge transfers and amplitudes (Figure 7F). Furthermore, when we recorded GTCs from spinal slices obtained from TLR4 knockout mice, we found that GTCs remained unchanged after bath-perfusion of paclitaxel (1 ng/ml) (Figure 7G). Together, these data indicate that paclitaxel reduces glial glutamate transporter activities through activating TLR4.

## Discussion

Our study is the first to reveal that paclitaxel induces acute pain through activating TLR4. Specifically, we found that low levels of paclitaxel penetrates into the CSF in rats receiving i.v. injection of paclitaxel. Intrathecal injection of paclitaxel induces mechanical allodynia in a dose-dependent manner. We also provide evidence that paclitaxel induces activation of TLR4 in the spinal dorsal horn and dorsal root ganglions. Activation of TLR4 by paclitaxel increases glutamatergic synaptic activities and reduces glial glutamate transporter activities. The cellular and molecular signaling pathways revealed in this study could provide strategies for the development of analgesics and management of P-APS in patients.

### Acute pain induced by paclitaxel treatment

P-APS is a significant morbidity in patients receiving paclitaxel treatment, with an incidence of 88% in patients treated with paclitaxel at a dose of more than 175 mg/m^2^ in the first cycle [1,2]. The pain in patients is most prominent in the lower extremities [1,2]. P-APS in patients receiving paclitaxel at a dose of more than 175 mg/m^2^ usually occurs within 1–3 days after drug administration and resolves within 7 days [1]. In this study, we found that paclitaxel induces acute pain in rodents in a dose-dependent manner. The onset and duration of acute pain in rodents are shorter than those for P-APS in humans, which may reflect a difference in species and the low taxol doses used in this study. Nevertheless, our data presents the first evidence for the direct action by paclitaxel on spinal nociceptive sensory processing. The doses of 2 mg/kg (which equals 14.06 mg/m^2^) and 5 mg/kg (which equals 35.16 mg/m^2^) [61,62] used in this study are moderate doses, which are within the dose range widely used by many labs for the study of paclitaxel-induced neuropathic pain in rodents [28,63-67]. Further, since the dose of taxol used in clinics ranges from 15 mg/m^2^ up to 825 mg/m^2^ [68-72], the doses used in this study are a conservative simulation of clinically relevant doses.

### Paclitaxel penetrating into the spinal dorsal horn is a critical event leading to the development of acute pain following paclitaxel injection

Currently, mechanisms underlying P-APS remain unknown. Our current study provides evidence that paclitaxel penetrating into the spinal dorsal horn contributes to the development of acute pain following paclitaxel injection in animals. Using liquid chromatography tandem mass spectrometry, we, for the first time, show that rats receiving a single i.v. paclitaxel (2 mg/kg) injection had low levels of paclitaxel penetrating into the CSF and spinal dorsal horn between 2 to 4 hrs after the paclitaxel injection. These levels went down significantly at 24 hrs post injection. These data are in agreement with previous studies in human cerebral spinal fluid and rodent spinal cord and brain. For example, patients have paclitaxel levels in the CSF between 4.25 and 70.88 ng/ml at 3.25 to 5 hrs following intravenous injection of paclitaxel (90–200 mg/m^2^) [16], and 2.98 to 49.01 ng/ml at 0.5 hr after intravenous injection of paclitaxel (175 mg/m^2^) [17]. Paclitaxel levels in the spinal cord of rats are between 5.9 to 17.3 ng/g on day 11 after rats receiving intravenous application of paclitaxel 5 mg/kg/day on days 1, 2, 3, 9 and 10 [18]. Despite the existence of paclitaxel in the CNS, no studies have been reported on the impact of paclitaxel on CNS functions. Here, we demonstrated that topical application of paclitaxel in the intrathecal space induces mechanical allodynia in a dose-dependent manner.

Among many factors leading to aberrant activation of neurons in the pain signaling pathway, excessive activation of glutamate receptors in the spinal dorsal horn is a key factor [21,22]. The activation of glutamate receptors is determined by three essential factors: the amount of synaptically released glutamate, the rate at which glutamate is removed by glutamate transporters, and the properties and number of postsynaptic glutamate receptors [73,74]. Glial glutamate transporters account for over 90% of all CNS synaptic glutamate uptake [25]. Our present study found that paclitaxel in the spinal dorsal horn can enhance glutamate receptor activation by altering all of these three factors. Paclitaxel at a concentration as low as 1 ng/ml (which is at the low end of the paclitaxel concentration range found in the CSF 2 to 4 hrs after paclitaxel injection) significantly increases glutamatergic synaptic activities by facilitating presynaptic glutamate release and increasing glutamate receptor activities at the postsynaptic neurons in the spinal dorsal horn (Figure 5). Paclitaxel at this concentration also significantly reduced glial glutamate transporter activities in the same area (Figures 6 and 7). Further, we also found that topical application of paclitaxel (2 ng/ml) on the surface of the intact spinal cord causes the reduction of glutamate transporter activities in the spinal dorsal horn (Figure 6). These findings indicate that paclitaxel penetrating into the spinal dorsal horn and the subsequent enhanced activation of glutamate receptors as well as the reduction of glial glutamate transporters play an important role in the acute pain induced by paclitaxel. Further studies are warranted to investigate how glutamatergic synaptic activities are enhanced by paclitaxel.

### Paclitaxel induces acute pain through activating TLR4

Numerous studies have shown that activation of TLR4 is a critical component contributing to the genesis of pathologic pain. For example, intrathecal or peritoneal injection of the TLR4 agonist LPS induces allodynia in mice [75] and rats [76,77]. Pharmacological blocking or gene knockout of TLR4 attenuates both thermal and mechanical allodynia in mice with neuropathic pain induced by nerve injury, and prevents activation of the transcription factor NF-kB and the over-production of TNFα and IL-1β in the spinal cord [13-15]. Paclitaxel has LPS-mimetic activity causing the activation of TLR4. For example, exposing murine peritoneal macrophages [8], breast cancer cell lines [9], and human monocytes [7] to paclitaxel causes activation of TLR4 and release of TNFα and IL-1β. Currently no studies have been reported regarding the direct activation of TLR4 in the nervous system by paclitaxel and its functional implications. We collected several lines of evidence supporting the critical role of TLR4 activation in the genesis of paclitaxel induced acute pain: a). TLR4 in the spinal dorsal horn and dorsal root ganglions is activated in animals receiving paclitaxel at a dose that simulates the low end of doses used clinically (Figure 3); b). the paclitaxel-induced acute allodynia was abolished in TLR4 knockout mice or by intrathecal injections of a TLR4 antagonist in rats (Figure 4); c). TLR4 mediates the enhanced glutamatergic synaptic activities (Figure 5), as well as the reduced glial GT activities (Figure 7) induced by paclitaxel in the spinal dorsal horn.

## Conclusions

Our study for the first time reveals a mechanism underlying paclitaxel induced acute pain in animals. We identify that activation of TLR4 in the spinal dorsal horn and dorsal root ganglions, increased glutamatergic synaptic activities, and reduced glial glutamate transporter activities in the spinal dorsal horn are critical events in the genesis of the paclitaxel-induced acute pain in animals. Thus, preventing or reversing these abnormalities could potentially prevent and attenuate P-APS in patients.

## Methods and materials

### Animals

Adult male (220 to 260 g) Sprague Dawley rats and 6–8 week old male wild-type mice (strain: C57BL/6J), and TLR4 knockout mice (Strain: B10scN-Tlr4lps-del/JthJ) were used. All experiments were approved by the Institutional Animal Care and Use Committee at the University of Georgia and were fully compliant with the National Institutes of Health Guidelines for the Use and Care of Laboratory Animals.

### Drug administration

Paclitaxel (2 mg/kg, or 5 mg/kg) was injected into rats through the tail vein to mimic the i.v. administration of paclitaxel used in the clinic. Mice received paclitaxel (2 mg/kg) through intraperitoneal (i.p.) injection. Paclitaxel (Taxol, Bristol-Myers Squib, 6 mg/ml in Cremophor EL and dehydrated ethanol) was diluted with saline to make up a volume of 1 ml for rats or 0.25 ml for mice for injection. Vehicle was composed of the same amounts of Cremophor EL and dehydrated ethanol (Sigma Chemicals, St. Louis, MO, USA) diluted with saline to the same volume. The experimenter was blind to the type of drugs injected to the animal. Intrathecal drug administration was made either through a pre-implanted intrathecal catheter or lumbar puncture. To make the implantation of a intrathecal catheter, a polyethylene (PE-10) catheter that ended at the spinal L4 segment was intrathecally placed following the technique previously described [78]. Briefly, rats were anesthetized under isoflurane (2-3%) and the atlanto-occipital membrane was exposed by dissection. A PE-10 catheter was carefully advanced through an opening in the atlanto-occipital membrane to the lumbar enlargement. The wound was then closed in layers. The animals were allowed to recover for 7 days before behavioral tests were conducted. Following behavioral experiments, rats were intrathecally injected with 50 μl of 2% lidocaine. If hind paw paralysis did not ensue, rats were omitted from the experiment. For drug administration through the lumbar puncture, drugs were injected into the intrathecal space at the L5-L6 lumbar interspace in rats anesthetized with 2% isoflurane using a 0.5-inch 30-gauge needle connected to a Hamilton syringe as described previously [79,80].

### Behavioral tests

#### Measurements of mechanical thresholds of hind paw withdrawal responses

Behavioral tests were conducted in a quiet room with the room temperature at 22°C [81,82]. To test possible changes in mechanical sensitivity after a paclitaxel injection, rats or mice were placed on a wire mesh, loosely restrained under a plexiglass cage (12 × 20 × 15 cm^3^) and allowed to acclimate for at least 30 min for rats and 1.5 h for mice. A series of von Frey monofilaments (bending force from 0.07 to 26.00 g) were tested in ascending order to generate response-frequency functions for each animal. Each von Frey filament was applied 5 times to the mid-plantar area of each hind paw from beneath for about 1 s. The response-frequency [(number of withdrawal responses of both hind paws/10) × 100%] for each von Frey filament was determined. Withdrawal response mechanical threshold was defined as the lowest force filament that evoked a 50% or greater response-frequency. This value was later averaged across all animals in each group to yield the group response threshold [44,82].

#### Burrowing behavior assessments

Protocols established by others for assessments of burrowing behaviors [33,34] were used. Rats were trained to burrow based on a previous protocol [33,34]. For baseline measurements of burrowing activities, individual rats were placed in an empty cage for 30 min for acclimation. A tube (32 cm × 10 cm) filled with 2500 g of sea gravel was then placed in the cage for 1 hr and burrowing was conducted during the light phase. Burrowing activities were measured by weighing the remaining gravel in the tube after each 1 hr burrowing session. The percentage of gravel displaced was calculated and used for statistical analysis. The following day after baseline measurements, rats were randomly assigned to receive either i.v. injections of taxol or vehicle. Burrowing activities at different time points after the injection were determined.

### Measurements of paclitaxel concentrations in CSF and spinal dorsal horn using liquid chromatography tandem mass spectrometry

At 2, 4 and 24 h after paclitaxel (2 mg/kg) was injected into rats through the tail vein, CSF was obtained through intracisternal puncture from rats anesthetized via isoflurane inhalation. The dorsal half of the L4-L5 spinal segments was collected at the same time point. Paclitaxel concentrations in CSF and in the spinal dorsal horn were measured using liquid chromatography tandem mass spectrometry techniques developed in our lab, which were described in detail previously [83].

### Spinal slice preparations, recording and analysis of miniature excitatory postsynaptic currents (mEPSCs) from neurons and glial glutamate transporter currents (GTCs) from astrocytes in the spinal dorsal horn

#### Spinal slice preparations

Transverse rat or mouse spinal cord slices (400 μm) of the L4-L5 segments were prepared as previously described [48,49]. Briefly, animals were deeply anesthetized via isoflurane inhalation. Surgery was performed to expose and remove the spinal lumbar enlargement segment. The lumbar spinal cord section was then placed in ice-cold sucrose artificial cerebrospinal fluid pre-saturated with 95% O_2_ and 5% CO_2_. The sucrose aCSF contained 234 mM sucrose, 3.6 mM KCl, 1.2 mM MgCl_2_, 2.5 mM CaCl_2_, 1.2 mM NaH_2_PO_4_, 12.0 mM glucose, and 25.0 mM NaHCO_3_. The pia-arachnoid membrane was removed from the section. The L4-L5 spinal segments was attached with cyanoacrylate glue to a cutting support, which was then glued onto the stage of a vibratome (Series 1000, Technical Products International, St. Louis, MO). Transverse spinal cord slices were cut in the ice-cold sucrose aCSF and then pre-incubated in Krebs solution oxygenated with 95% O_2_ and 5% CO_2_ at 35°C. The Krebs solution contained: 117.0 mM NaCl, 3.6 mM KCl, 1.2 mM MgCl_2_, 2.5 mM CaCl_2_, 1.2 mM NaH_2_PO_4_, 11.0 mM glucose, and 25.0 mM NaHCO_3_ at 35°C.

#### Recordings of mEPSCs

Following pre-incubation, a single slice was placed in the recording chamber (volume, 1.5 ml), perfused with Krebs solution at 35°C, and saturated with 95% O_2_ and 5% CO_2_. Borosilicate glass recording electrodes (resistance, 3–5 MΩ) were pulled and filled with an internal solution containing 135 mM potassium-gluconate; 5.0 mM KCl; 2.0 mM MgCl_2_; 0.5 mM CaCl_2_; 5.0 mM HEPES; 5.0 mM EGTA; 5.0 mM ATP-Mg; 0.5 mM Na-GTP; 10 mM QX-314. Live dorsal horn neurons in the spinal lamina I and outer lamina II (IIo) were visualized using a microscope system and approached using a three-dimensional motorized manipulator (Sutter Instrument, Novato, CA, USA), and whole-cell configurations were established by applying moderate negative pressure after electrode contact [84]. Recordings of mEPSCs were made from neurons receiving monosynaptic input from the primary afferents using the criteria established previously [81,85]. Miniature EPSCs were recorded in the presence of tetrodotoxin (TTX, 1 μM), bicuculline (10 μM), and strychnine (5 μM) in the external solution to block GABA_A_, and glycine receptors at a membrane potential at −70 mV.

#### Recordings of glutamate transporter currents (GTCs)

GTCs were recorded from male wild-type mice and TLR4 knockout mice. Other than deletion of the TLR4 gene, TLR4 knockout mice (B10scN-Tlr4lps-del/JthJ) have a similar genomic background as C57BL/6J mice. The mouse spinal slice was placed in a recording chamber perfused with Krebs solution. Astrocytes in the spinal dorsal horn laminae I and II were first labeled by the astrocyte specific dye, sulforhodomine 101 (100 μM) [58], which was pressure-injected into the spinal slice through a pipette with a picospritzer [29,58,59]. The identified astrocyte was patched using borosilicate glass recording electrodes (resistance, 4–6 MΩ) filled with (in mM) 145 potassium-gluconate, 5 NaCl, 1 MgCl_2_, 0.2 EGTA, 10 HEPES, 2 Mg-ATP and 0.1 Na-GTP (pH 7.3, 290 – 300 mOsm) [51,86]. GTCs were recorded at a holding potential of −80 mV in voltage clamp mode in the presence of blockers of GABA_A_ receptor (10 μM bicuculline), glycine receptor (5 μM strychnine), AMPA/kainate receptors (10 μM DNQX), NMDA receptor (25 μM D-AP5), and tetrodotoxin (1 μM) at 35°C [51,56]. GTCs were evoked by puffing 50 μM L-glutamate onto the recorded astrocyte through a glass pipette connected to a Picospritzer controlled by a computer.

mEPSCs and GTCs were recorded using Axopatch 700B amplifiers, digitized at 10 kHz, and analyzed off-line. Access resistance within the range of 10–20 MΩ was monitored continuously throughout the experiments. The recording was abandoned when the access resistance changed more than 20%. The frequency and amplitude of mEPSCs from 3 min before, during, and after the perfusion of tested drugs were analyzed and averaged using a peak detection program (MiniAnalysis; Synaptosoft Inc., Decatur, GA). Four sweeps of GTCs were averaged and the mean amplitude and charge transfer of GTCs [87] were measured. All the drugs were applied through bath-perfusion unless otherwise indicated.

### In vitro measurement of glutamate uptake activity

Synaptosome preparations were prepared from the spinal tissue. The glutamate uptake activity in the synaptosome preparation was measured according to previous publications [51,88]. To investigate the effects of paclitaxel on glutamate transporter activities, the L4–L5 spinal cord was exposed by laminectomy and the spinal dura was excised in rats anesthetized with urethane (1.3–1.5 g/kg, i.p). The heart rate, breathing, and core temperature of the animals were constantly monitored and maintained within normal limits [82]. Paclitaxel was applied onto the L4-L5 spinal segments through a piece of cotton soaked with paclitaxel (concentration: 2 ng/ml) in aCSF at 35°C for 30 min. Rats in the control group receiving vehicles in the same fashion. Immediately after the treatment, the dorsal half of the L4-L5 spinal segments was isolated. Synaptosome preparations were prepared immediately after the spinal tissue was isolated according to the protocol published [50,51]. The spinal tissue was homogenized in ice-cold buffer solution containing: 0.5 mM EDTA, 0.5 mM EGTA, 0.2 mM phenylmethylsulfonyl fluoride, 0.32 M sucrose, 5 μg/ml pepstatin, 5 μg/ml aprotinin, 20 μg/ml trypsin inhibitor, 4 μg/ml leupeptin, and 0.01 M phosphate-buffered saline. The homogenates were centrifuged at 15,000 rpm for 10 min at 4°C, and the supernatant collected. The remaining pellets were resuspended in the same buffer solution and re-centrifuged at 15,000 rpm for 10 min at 4°C. The two supernatants were combined and centrifuged again at 13,000 rpm for 10 min at 4°C to obtain the synaptosomal pellets, which contained both neuronal and glial glutamate transporters [89]. The synaptosomal pellets were suspended in Locke’s buffer. The glutamate uptake activity was determined by incubating the synaptosome preparation with a solution containing [^3^H] L-glutamic acid (0.4 μCi/mmol) at 37°C for 5 min. The reaction was terminated by filtering the synaptosomes through a Whatman GF/B filter presoaked with the same buffer solution. The filter was then transferred to a vial containing scintillation cocktail and the radioactivity, which reflects glutamate uptake activities, in the final samples was measured by a liquid scintillation counter (Beckman, LS6500).

### Western blot experiments

Tissues used for Western blotting were either from intact animals that received i.v. injection of paclitaxel (2 mg/kg) or vehicle 4 hrs earlier, or intrathecal injection of paclitaxel (20 ng) or vehicle 1 hr earlier, or spinal slices that were incubated with paclitaxel (1 ng/ml) or vehicles for 15 min. Animals were deeply anesthetized with urethane (1.3–1.5 g/kg, i.p.). The L4-L5 spinal segments and dorsal root ganglions were exposed by surgery and removed from the rats. Rat spinal slices of the spinal L4-L5 segments were obtained in the same way as those for electrophysiological experiments described above. Spinal slices were incubated with vehicles or paclitaxel (1 ng/ml) in aCSF bubbled with 95% O_2_ and 5% CO_2_ at 35°C for 15 min. The dorsal halves of the spinal cord and dorsal root ganglions were then isolated and quickly frozen in liquid nitrogen and stored at −80°C for later use. The frozen tissues were homogenized with a hand-held pellet pestle in lysis buffer (50 mM Tris, pH 7.5, 150 mM NaCl, 1 mM EDTA, 0.1% SDS, 1% Deoxycholic acid, 2 mM orthovanadate, 100 mM NaF, 1% Triton X-100, 0.5 mM phenylmethylsulfonyl fluoride, 20 μM leupeptin, 100 IU ml^−1^ aprotinin) for 0.5 hr at 37°C. Homogenates were then centrifuged at 14000 × g for 20 min at 4°C and the supernatant were collected. For measuring protein GLT-1 and GLAST expressions in the plasma membrane and cytosol, the tissue was fractionated into cytosolic and membrane fractions with the cytoplasmic, nuclear, and membrane compartmental protein extraction kit (Biochain Institute, Inc.). Protein concentrations were determined using bicinchoninic acid (kit from Pierce). Protein samples (40 μg) were electrophoresed in 10% SDS polyacrylamide gels and transferred to polyvinylidene difluoride membranes (Millipore, Bedford, MA). The membranes were blocked with 5% milk and incubated overnight at 4°C with primary antibodies against phospho-TLR4/CD284 pTyr674 (1:1000, Thermo Scientific Pierce Antibodies), TLR4 (1:1000, Abcam, Cambridge, MA), GLT-1 (1:1000, Millipore, Bedford, MA), GLAST (1: 2000, Millipore, Bedford, MA) or a monoclonal mouse anti-β-actin (1:2000, Sigma-Aldrich, St. Louis, USA) primary antibody as a loading control. The cytosolic and membrane fractions were checked for specificity by Western blotting with anti-tubulin (1:200), anti-EGFR (1:200; Santa Cruz Biotechnology). Then the blots were incubated for 1 hr at room temperature with a corresponding HRP-conjugated secondary antibody (1:5000; Santa Cruz Biotechnology, CA, USA), visualized in ECL solution (SuperSignal West Pico Chemiluminescent Substrate, Pierce, Rockford, IL, USA) for 1 min, and exposed onto FluorChem HD2 System. The intensity of immunoreactive bands was quantified using ImageJ 1.46 software (NIH). Results were expressed as the ratio to control protein.

### Single-cell reverse transcription-PCR (RT-PCR)

Single-cell RT-PCR was performed as described previously [90,91]. Briefly, after electrophysiological recordings, the cell which had been labeled with sulforhodomine 101 for astrocytes [58] was harvested into a fresh “patch” pipette under fluorescence microscopy with a heat-polished tip opening diameter of about 20 μm, which was filled with pipette solution (6 μl) supplemented with 3 U recombinant ribonuclease inhibitor (RNasin; Promega, Madison,WI). The cell was gently transferred into a reaction tube (final volume about 10 μl) containing 3.5 μl reaction mix, reverse transcriptase buffer (Qiagen), deoxyribonucleotide triphosphates (dNTPs, concentration: 4 × 250 μM; Applied Biosystems, Weiterstadt, Germany), random hexanucleotide primer (50 μM; Roche, Mannheim, Germany), 20 U RNasin (Promega), and 0.5 μl Sensiscript reverse transcriptase (Qiagen). The reaction was performed at 37°C (1 hour). The cDNA of a single cell was amplified using Sensiscript RT kit (Qiagen). Glial fibrillary acidic protein were used to identify the harvested cell type. PCR amplification was performed using the primers shown in Table 1. All PCR amplifications were performed with nested primers. The first round of PCR was performed in 50 μl of PCR buffer containing 0.2 mM dNTPs, 0.2 μM “outer” primers, 5 μl of RT product and 0.2 μl of platinum TaqDNA polymerase (Invitrogen), 1.5 mM MgCl_2_. Samples were heated to 95°C for 5 min. Each cycle consisted of denaturation at 95°C for 40 s, annealing at 55°C for 40 s, and elongation at 72°C for 40 s. Forty-two cycles were performed with a programmable thermocycler (Bio-Rad). The reaction was completed with 7 min of final elongation. For the second round of amplification, the reaction buffer (20 μl) contained 0.2 mM dNTPs, 0.2 μM “inner” primers, 5 μl of the first round PCR products, 0.1 μl of platinum TaqDNA polymerase and 1.5 mM MgCl_2_. The second PCR condition consisted of denaturation at 95°C for 30 s, annealing at 55°C for 40 s, elongation at 72°C for 40 s for 35 cycles, and the reaction was completed with 7 min of final elongation and subsequent cooling to 4°C until analysis. A negative control was obtained from pipettes that did not have cell contents but were submerged in the bath solution. PCR products were analyzed on 2% agarose gels.

**Table 1:** **List of DNA primer sequences designed for single-cell RT-PCR**

GFAP (371 bp, 218 bp)^a^	CAGAGCGAGCCTATGCTAAA	GCCTATGCTAAAGGTTAGGTTGTA	NM_001131020
	CGTCCAGAGGGAACTAACTAAC	AGCACTGAAGTGAAGCAATAGA	
GAPDH (367 bp, 313 bp)^a^	AGCCTCGTCCCGTAGACAAAA	TGAAGGTCGGTGTGAACGAATT	XM_001473623
	TTTTGGCTCCACCCCTTCA	GCTTTCTCCATGGTGGTGAAGA	

^a^(n, n) indicates product size obtained from outer and inner primers, respectively.

### Materials

Bicuculline, strychnine, tetrodotoxin, 6,7-dinitroquinoxaline-2,3-dione (DNQX), L-glutamic acid were obtained from Sigma (St. Louis, MO, USA). (2S,3S)-3-[3-[4-(Trifluoromethyl)benzoylamino]benzyloxy]aspartate (TFB-TBOA), D-2-amino-5-phosphonopentanoate (D-AP5) were obtained from Tocris Bioscience (Minneapolis, MN, USA). Sulforhodamine 101 and LPS-RS were purchased from Invitrogen (San Diego, CA). [^3^H] L-glutamic acid was obtained from Perkin Elmer.

### Data analysis

All data are presented as the mean ± S.E. The statistical differences were determined using Student’s *t*-test (paired *t*-test for data obtained within the same group; non-paired *t*-test for data obtained from different groups). A *P* value less than 0.05 was considered statistically significant.
